# CD26 Is Differentially Expressed throughout the Life Cycle of Infantile Hemangiomas and Characterizes the Proliferative Phase

**DOI:** 10.3390/ijms25189760

**Published:** 2024-09-10

**Authors:** Bruno Lorusso, Antonella Nogara, Rodanthi Fioretzaki, Emilia Corradini, Roberta Bove, Giovanni Roti, Andrea Gherli, Anna Montanaro, Gregorio Monica, Filippo Cavazzini, Sabrina Bonomini, Gallia Graiani, Enrico Maria Silini, Letizia Gnetti, Francesco Paolo Pilato, Giuseppe Cerasoli, Federico Quaini, Costanza Anna Maria Lagrasta

**Affiliations:** 1 Department of Medicine and Surgery, University of Parma, 43126 Parma, Italy; brunolorusso@gmail.com (B.L.); antonella_nogara@libero.it (A.N.); rodanthifioretzaki@gmail.com (R.F.); emilia.corradini@unipr.it (E.C.); roberta.bove@studenti.unipr.it (R.B.); giovanni.roti@unipr.it (G.R.); andrea.gherli@unipr.it (A.G.); anna.montanaro@unipr.it (A.M.); gregorio.monica@unipr.it (G.M.); filippo.cavazzini@studenti.unipr.it (F.C.); federico.quaini@unipr.it (F.Q.); 2 Department of Medical Oncology, Metaxa Cancer Hospital of Piraeus, 185 37 Piraeus, Greece; 3 Translational Hematology and Chemogenomics (THEC), University of Parma, 43126 Parma, Italy; 4 Hematology and BMT Unit, University Hospital of Parma, 43126 Parma, Italy; sabrinabonomini@yahoo.it; 5 Center of Dental Medicine, University of Parma, 43126 Parma, Italy; gallia.graiani@unipr.it; 6 Pathology Section, University Hospital of Parma, 43126 Parma, Italy; enricomaria.silini@unipr.it (E.M.S.); letyg70@hotmail.com (L.G.); pillogro@gmail.com (F.P.P.); 7 Pediatric Surgery, Ospedale dei Bambini of Parma, University Hospital of Parma, 43126 Parma, Italy; gcerasoli@yahoo.it

**Keywords:** proliferative infantile hemangioma, immunohistochemistry, CD26, CD133, endothelial cells, proliferation, p21, ki67, apoptosis, cell cycle

## Abstract

Infantile hemangiomas (IHs) are benign vascular neoplasms of childhood (prevalence 5–10%) due to the abnormal proliferation of endothelial cells. IHs are characterized by a peculiar natural life cycle enclosing three phases: proliferative (≤12 months), involuting (≥13 months), and involuted (up to 4–7 years). The mechanisms underlying this neoplastic disease still remain uncovered. Twenty-seven IH tissue specimens (15 proliferative and 12 involuting) were subjected to hematoxylin and eosin staining and a panel of diagnostic markers by immunohistochemistry. WT1, nestin, CD133, and CD26 were also analyzed. Moreover, CD31^pos^/CD26^pos^ proliferative hemangioma–derived endothelial cells (Hem-ECs) were freshly isolated, exposed to vildagliptin (a DPP-IV/CD26 inhibitor), and tested for cell survival and proliferation by MTT assay, FACS analysis, and Western blot assay. All IHs displayed positive CD31, GLUT1, WT1, and nestin immunostaining but were negative for D2-40. Increased endothelial cell proliferation in IH samples was documented by ki67 labeling. All endothelia of proliferative IHs were positive for CD26 (100%), while only 10 expressed CD133 (66.6%). Surprisingly, seven involuting IH samples (58.3%) exhibited coexisting proliferative and involuting aspects in the same hemangiomatous lesion. Importantly, proliferative areas were characterized by CD26 immunolabeling, at variance from involuting sites that were always CD26 negative. Finally, in vitro DPP-IV pharmacological inhibition by vildagliptin significantly reduced Hem-ECs proliferation through the modulation of ki67 and induced cell cycle arrest associated with the upregulation of p21 protein expression. Taken together, our findings suggest that CD26 might represent a reliable biomarker to detect proliferative sites and unveil non-regressive IHs after a 12-month life cycle.

## 1. Introduction

Infantile hemangiomas (IHs) are benign vascular neoplasms of childhood (prevalence 5–10%) resulting from the abnormal proliferation of endothelial cells. Prematurity, low birth weight, Caucasian ethnicity, and female gender represent risk factors for the disease. IHs may appear weeks after birth and display a distinctive life cycle that can be separated into three phases: proliferative (≤12 months of age; rapid growth during the first few weeks or months of life), involuting (plateau period begins at approximately ≥13 months of age), and involuted (up to 4–7 years). At histologic analysis, the proliferative phase shows numerous vessels and capillaries with collapsed lumen and cuboid lining endothelial cells. In the involuting phase, capillary and vessel lumens are wide and lined by flat endothelial cells. The involuted phase displays a characteristic fibro-fatty residuum [1,2].

Although about 85–90% of all IHs undergo spontaneous involution, a small percentage needs intervention to prevent or treat complications such as functional impairment, disfigurement, scarring, local painfulness, ulceration, or for a continuous, inexplicable growth. Since 2008, the management of IHs has undergone significant change with the introduction of systemic or topical treatment with beta-blockers [3,4,5].

Initially classified in 1982 to distinguish IH from other vascular tumors and malformations, in the last few decades, experimental and pathologic data have been accumulated to clarify the origin of hemangioma endothelium and improve the histological classification in order to provide more accurate diagnostic criteria and assess the validity of therapeutic approaches [6].

The pathogenesis of the disease remains unknown, and current hypotheses have not been fully validated [7]. The most accepted pathogenetic mechanisms are hypoxic stress, involvement of the renal-angiotensin system, and recruitment of endothelial progenitor cells in hemangiomatous lesions [7,8,9,10].

Hemangioma endothelial cells in all phases express the pan-endothelial markers CD31/PECAM-1 (Platelet Endothelial Cell Adhesion Molecule-1) and GLUT1, a glucose transporter normally present in the microvascular endothelium of the blood-brain barrier, retina, placenta, and red blood cell membrane, but not in normal skin [11,12,13].

North et al. described GLUT1 as a specific marker of IHs in all developmental phases and absent in other vascular lesions, such as congenital hemangioma, vascular malformations, pyogenic granulomas, granulation tissue, and several types of hemangioendotheliomas [14].

The immunohistochemical characterization of IH typically displays negative staining for D2-40 (a specific lymphatic endothelial marker) and increased proliferation index by ki67 staining of IH endothelial cells. In addition, the identification of markers associated with tumor angiogenesis as Wilms’ tumor 1 protein (WT1) and nestin and cancer stem cell lineages (CD133 or prominin) has also been reported [15,16,17,18,19].

Specifically, membrane glycoprotein CD133, discovered in 1997, alone or in combination with other markers, is one of the most well-characterized stem cells (SCs)-associated antigens in various tissues and in cancer stem cells (CSCs) from different neoplasms, including vascular tumors [19,20,21,22,23,24,25,26]. Yu et al., in 2004, revealed CD133 to be expressed by the endothelium of proliferative IH vessels [19].

Focus has been recently on CD26, a protein with known dipeptidyl-peptidase IV (DPP-IV) activity [27]. DPP-IV/CD26 has been closely related to the oncogenic process as a marker of malignancy in colorectal cancer, pleural mesothelioma, hepatocellular carcinoma, and hematologic disorders, with potential implication on stemness pathways [28,29,30,31,32,33]. However, the specific role of CD26 on cancer stemness is still contradictory and may vary according to cancer-specific contexts [34].

In the present study, in parallel with typical immunohistochemical markers used for routine diagnostics, we investigated whether CD26 could be differentially expressed according to the life cycle of IH. Furthermore, we explored the functional role of DPP-IV/CD26 on proliferative hemangioma–derived endothelial cells in vitro.

## 2. Results

### 2.1. Immunohistochemical Analysis of Infantile Hemangioma

**Figure 1:**
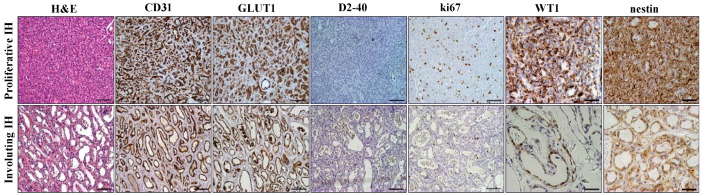
Histologic and immunohistochemical analysis of infantile hemangioma. Representative images of H&E and immunohistochemically stained sections from proliferative (**upper panel**, i.e., 5 months) and involuting (**lower panel**, i.e., 17 months) infantile hemangioma (IH) tissue samples. The expression of CD31, GLUT1, and D2-40 commonly used for routine diagnostics is illustrated by immunoperoxidase (brownish). Increased ki67, WT1, and nestin labeling of vascular cells in proliferative IH can be appreciated. Scale bars = 100 µm except for WT1 and nestin (50 µm).

The clinical diagnosis was confirmed in all cases by the histologic and immunohistochemical analysis. Endothelial cells lining the vascular network were strongly positive for CD31 and GLUT1, while they were negative for D2-40, which labeled lymphatic vessels in the spared areas. We also documented a high proliferation rate of endothelial cells by ki67 immunostaining (Figure 1). In agreement with literature reports, the endothelial cells and pericytes of all proliferative IHs displayed strong and diffuse WT1 and nestin cytoplasmic staining [15,16]. Conversely, involuted IHs showed a granular-like pattern of WT1 and a weak nestin signal (Figure 1).

### 2.2. CD26 and CD133 Expression

**Figure 2:**
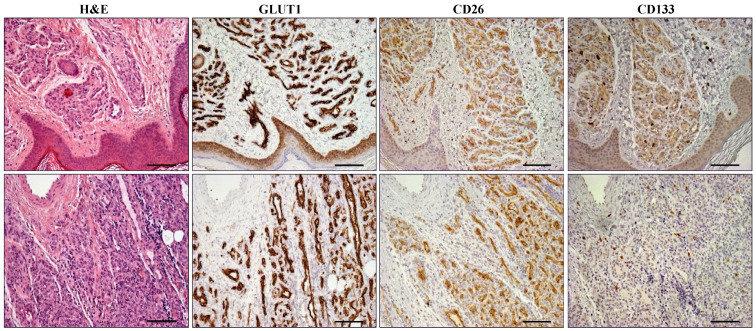
Proliferative infantile hemangioma. Representative H&E and immunohistochemically stained serial sections of proliferative infantile hemangiomas from a 5- (**upper panel**) and a 3- (**lower panel**) month-old patient. Numerous vessels and capillaries with collapsed lumen and cuboid lining endothelial cells labelled by GLUT1 and CD26 are apparent in the same sampled area. Positive CD133 staining was detected only on the upper case. Scale bars = 100 µm.

We next examined the expression of CD26 and CD133 on immunohistochemical preparations from each subset of IH samples. We observed that virtually 100% of ECs present in all proliferative IHs (≤12 months; n = 15) express CD26 (Figure 2). All endothelial cells in IH samples were uniformly stained by CD26, while they were negative in large or pre-existing vessels (Figure 2 and Figure S1). Of note, 5 out of 15 samples from proliferative IHs (3 to 10.5 months; mean = 7.2) were negative for CD133 staining (Figure 2).

**Figure 3:**
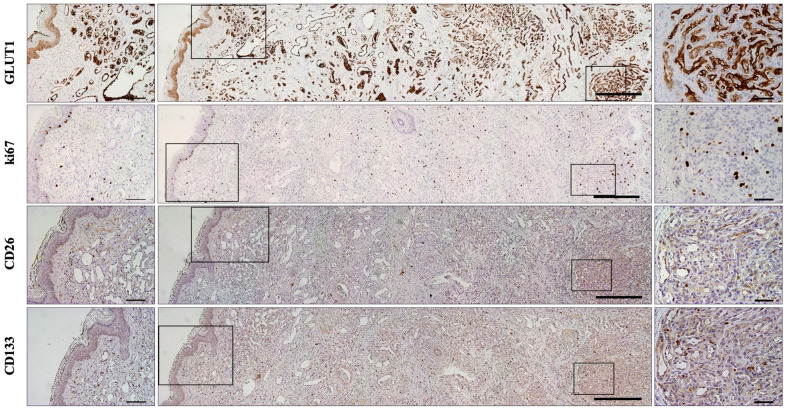
Concurrent proliferative and involuting phases in infantile hemangioma. Representative images of the immunohistochemical detection of distinguishable proliferative (**right**) and involuting (**left**) phases of infantile hemangioma on serial sections from the same 15-month-old case. The tissue areas inscribed by black squares are shown at higher magnification on the right and left images, respectively, to appreciate the different intensity of ki67, CD26, and CD133 labeling of vascular profiles in proliferative areas, while GLUT1 is uniformly expressed. Scale bars: **left panels** = 100 µm; **middle panels** = 400 µm; **right panels** = 50 µm.

**Figure 4:**
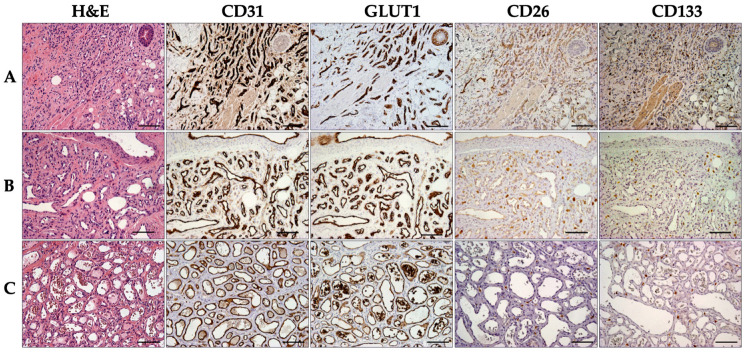
Involuting infantile hemangioma (IH). Representative images of the histological and immunohistochemical analysis performed on serial sections from three cases of IH. Sections were stained with H&E and labeled by immunoperoxidase to detect CD31, GLUT1, CD26, and CD133. (**A**) The hypervascularized tissue surrounding epithelial and smooth muscular structures displays positive CD26 and CD133 signals in a sample from a 15-month-old patient. (**B**) Tissue sections from a 37-month-old case documenting positive CD26 immunostaining and the lack of CD133 marker in proliferating vascular structures. (**C**) The involuting infantile hemangioma architecture from a 14-month-old patient is characterized by the absence of CD26 and CD133 immunostaining. In (**B**,**C**), the strong granular CD26 and CD133 signals in interstitial areas likely correspond to mast cells. (**A**–**C**) scale bars = 100 µm.

Intriguingly, among the involuting IHs group (≥13 months; n= 12), seven samples (58.3%; 13–37 months; mean = 21.3) exhibited a morphologically distinguishable mixed phase in which proliferative and involuting aspects coexisted in the same hemangiomatous lesion (Figure 3). Importantly, CD26 expression was detectable in these histologically recognized proliferative areas characterized by collapsed vessels and capillaries and cuboid endothelial cells. However, only three (42.8%; age 15–20 months; mean = 16.6) of the samples with circumscribed CD26 positive areas also showed CD133 immunostaining. Thus, CD133 was expressed in only 25% of the overall involuting hemangiomas. In contrast, peculiar involuting IH areas, consisting of wide vessel lumens covered by flat endothelial cells with prominent nuclei and surrounded by poor parenchymal cells, always lacked both CD133 and CD26 immunostaining (Figure 3 and Figure 4). The incidence of samples displaying CD26 or CD133 labeling of endothelial cells in tissue samples from proliferative and involuting IHs is reported in Table 1.

### 2.3. In Vitro Effects of DPP-IV Pharmacological Inhibition on Hem-ECs

Based on immunohistochemical observations, we investigated whether the in vitro pharmacological inhibition of DPP-IV affects the biological properties of Hem-ECs. 

**Figure 5:**
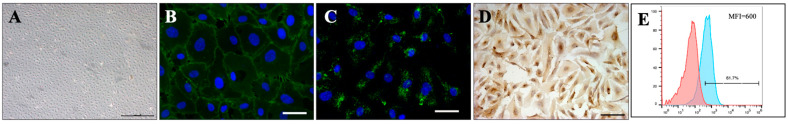
Morphologic and immunophenotypic characterization of isolated infantile hemangioma-derived endothelial cells (Hem-ECs). (**A**) Image from phase contrast microscopy illustrating a confluent monolayer of ECs, with typical cobblestone-like morphology, isolated and expanded from a proliferative IH tissue sample. The surface expression of the pan-endothelial marker CD31 (**B**) and the dot-like cytoplasmic labeling of von Willebrand factor (**C**) are shown in green by immunofluorescence. Nuclei (blue) are counterstained by DAPI. (**D**) GLUT1 expression in Hem-ECs is shown by immunoperoxidase (brownish). Nuclei are counterstained by light hematoxylin. Scale bars: (**A**) = 500 µm; (**B**,**C**) = 50 µm; (**D**) = 100 µm. (**E**) Representative diagram of the flow cytometric assay for the detection of CD26 on cultured Hem-ECs. The percentage and mean fluorescence intensity (MFI) of CD26 in ECs from a case of proliferative IH are reported. Red: unstained; Blue: mouse anti-human CD26-PE.

Thus, ECs isolated from surgical IH samples following enzymatic digestion and immunomagnetic sorting were cultured, expanded, and initially identified as endothelial cells according to morphologic criteria (Figure 5A). To ensure the purity of recovered cells, immunofluorescence was performed, documenting that the pan-endothelial markers CD31 (Figure 5B), von Willebrand factor (Figure 5C), and GLUT1 (Figure 5D) were consistently expressed on all examined cells. Then, to ascertain the presence of CD26 at the cellular level, we performed a flow cytometric analysis of cultured cells, documenting that more than 60% of Hem-ECs express CD26 in vitro (Figure 5E). 

**Figure 6:**
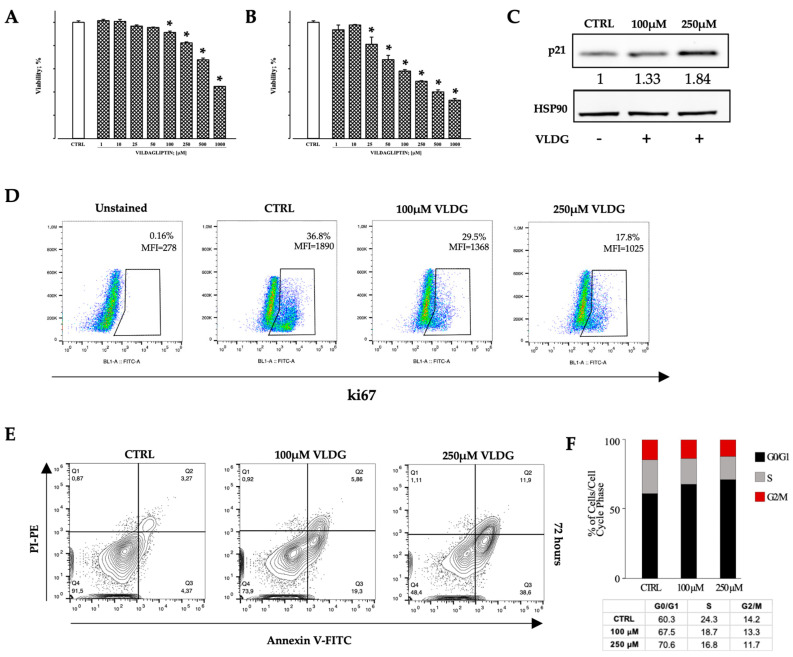
Impact of DPP-IV inhibition on infantile hemangioma-derived endothelial cells (Hem-ECs) in vitro. Hem-ECs treated with increasing vildagliptin (VLDG) concentrations for 24 h (**A**) and 72 h (**B**), respectively. Percent viability with respect to untreated cells (CTRL) is reported as mean ± standard error (SE); * *p* < 0.05 vs. control. Holm-Sidak’s test. (**C**) Immunoblotting analysis documenting a dose-dependent increase in p21 protein expression in Hem-ECs following VLDG exposure. HSP90 serves as the loading control. Densitometric values normalized vs. control (HSP90) are reported at the bottom of each p21 blot. (**D**) Flow cytometric analysis of Hem-EC proliferation. Gating strategy illustrating the percentage of ki67-positive untreated (CTRL) and VLDG-treated Hem-ECs. The density of scatter dot-plots in positive gate decreases in a dose-dependent manner. Mean fluorescent intensity (MFI) of ki67 labeling is also reported. (**E**) Apoptosis assay, as measured by the cytometric analysis of Annexin V, on Hem-ECs after 72 h exposure to 100 μM and 250 μM VLDG. (**F**) Cell cycle analysis of Hem-ECs after 72 h of VLDG treatment. The percentage of cells in the different phases of the cell cycle was calculated using the Watson Pragmatic Model of FlowJo (v10.8) software.

The potential inhibitory effect on cell viability of vildagliptin (VLDG, 1 to 1000 µM) was tested in Hem-ECs by MTT assay after 24 and 72 h of treatment. VLDG affected cell survival in a dose- and time-dependent manner (Figure 6A,B), reaching a statistically significant decrease at 100 µM concentration after 24 h and at 25 µM concentration after 72 h, respectively (*p* < 0.05). These data are also supported by the cytometric analysis of annexin V staining, documenting that VLDG induces Hem-ECs apoptosis in a dose-dependent manner (Figure 6E). 

As strongly suggested by the results from MTT, we explored cell-cycle-associated proteins in Hem-ECs exposed for 24 h to 100 µM and 250 µM VLDG. Specifically, a Western blot assay was employed to assess the p21 protein, a member of the Cip/Kip family of inhibitors of cell cycle progression. We observed that VLDG treatment up-regulated p21 protein expression in a dose-dependent manner (Figure 6C). In addition, the exposure to VLDG inhibited Hem-ECs proliferation as documented by flow cytometric assay showing a down-regulation of ki67, a nuclear protein expressed in G1, S, G2, and M phases of the cell cycle but absent in G0 (Figure 6D). 

Finally, the cell cycle analysis performed after 72 h of VLDG treatment documented that DPP-IV pharmacological inhibition is able to induce a dose-dependent G0/G1 cell cycle arrest in Hem-ECs (Figure 6F). 

## 3. Discussion

Although much knowledge has been achieved in IH and new therapeutic options have been introduced, leaving systemic glucocorticoids and surgical intervention as a second-line choice, several issues remain uncovered. In particular, the pathogenetic mechanisms underlying IH are largely unknown, and the hypotheses advanced so far are unable to fully elucidate the onset and evolution of the disease [2,5,7]. An additional confounding issue regarding the origin of this neoplastic disease is related to the contention that, in contrast to other vascular anomalies, to date, no germinal or somatic mutations have been definitely associated with IH [35,36].

In its early stages, IH displays cellular masses lacking distinct vascular architecture and expressing both mature and stemness-associated markers, underscoring high heterogeneity. A series of reports have documented the recruitment and presence of endothelial progenitor cells in hemangiomatous lesions following the detection of stem cell markers in vessels from IH samples [9,10,37,38]. The rising attention of the scientific community on these stemness-associated markers is attributable to the notion that the identification of CSCs represents an essential requisite to uncover the mechanisms underlying tumor initiation, progression, and self-renewal, as CSCs are essential key players in the pathogenesis and biology of neoplastic diseases, including vascular tumors [26,38].

In agreement with the latest 2018 ISSVA classification and previous reports, our investigation largely confirmed the neoplastic nature of IH through the documentation of overlapped and concurrent WT1 and nestin protein expression on IH vascular structures [14,15,39,40,41,42]. WT1 affects several downstream expression targets as nestin, a class IV intermediate filament protein originally described as a neuronal stem cell marker, which is expressed in newly formed blood vessels [43,44,45,46,47,48].

Additionally, in the last decade, several research groups have focused their attention on DPP-IV/CD26, a 110 kDa transmembrane glycoprotein capable of cleaving alanine- and proline-containing peptides at the penultimate position of bioactive properties as growth factors, cytokines, chemokines, neuropeptides, and vasoactive peptides. CD26 is expressed on numerous cell types of various organs, including the kidney, intestine, placenta, lung, liver, and prostate, and plays an important role in the immune response, autoimmunity, and diabetes [28,29,34]. 

In order to gain a better understanding of the underlying biology of IHs, our immunohistochemical analysis documented that the vascular endothelia of all proliferative IHs express surface CD26 at variance from involuting cases, suggesting that CD26 may play a role in tumor growth, proliferation, and evolution. 

Multiple studies have highlighted the key role of DPP-IV/CD26 in the progression of several human malignancies. High expression of CD26 on mesothelioma cells has been associated with increased proliferation, which can be reversed by humanized anti-CD26 monoclonal antibodies as experimentally shown in vivo and in vitro [34,49,50,51].

In hematologic malignancies, DPP-IV/CD26 activates and sustains the survival of acute and chronic myeloid leukemia stem cells [52,53], making this surface receptor a novel potentially targetable biomarker. In human colorectal carcinogenesis, CD26-positive CSCs promote proliferation and metastasis, in addition to affecting angiogenesis [31,54,55,56].

The novelty brought by the present investigation might reside in the potential implication of CD26 on the life cycle of infantile hemangiomas. Specifically, we observed that, at variance from involuting IH, proliferative hemangioma-derived endothelial cells express CD26 at tissue and cellular levels. In addition, in cases in which concurrent involuting and proliferative areas were detectable, CD26 appeared to selectively label discrete sites of vascular proliferation. A further involvement of DPP-IV/CD26 in the evolution of IH was supported by its in vitro pharmacological inhibition, resulting in significantly impaired Hem-EC survival and proliferation. Specifically, we selected vildagliptin, being the most employed DPP-IV inhibitor in patients affected by type 2 diabetes mellitus, whose safety profile has been clearly assessed [57].

The potential clinical implication of our findings might reside in the therapeutic effect of DPP-IV-targeting drugs on IH clinical outcome. However, this hypothesis should be tested only after a more comprehensive biological and experimental workout.

In conclusion, taken together, the results of the present investigation suggest CD26 is an effective biomarker of proliferative infantile hemangioma and is able to detect non-regressive sites within involuting IH. The potential clinical translation of our observations on CD26 expression in IH can be exploited to predict the true evolving phases of hemangiomatous lesions and to test its therapeutic targeting.

## 4. Materials and Methods

### 4.1. Patient Population and Tissue Sampling 

The patient population consisted of 27 children admitted to the Unit of Pediatric Surgery-Ospedale dei Bambini of Parma (from November 2018 to March 2022), enrolled after parental informed consent was received for the employment of biologic samples for research purposes. The patient cohort was divided by age so as to correspond to the conventional proliferative phase (n = 15; age 2.5 to 12 months, mean = 6.7; males = 6 and females = 9) and involuting phase (n = 12; age 13 to 37 months, mean = 19.7; males = 3 and females = 9) subsets. None of the children had received pharmacological treatment. Infantile hemangiomas were all single lesions and were located on the head/neck (n = 13; 48.1%), trunk (n = 9; 33.3%), vulvar lip (n = 4; 14.8%), among which two were ulcerated, probably due to nappy rubbing, or left thumb (n = 1; 3.7%). The procedure was approved by the Local Ethics Committee (129/2018/OSS/AOUPR) and in accordance with principles listed in the Helsinki declaration.

### 4.2. Immunohistochemical Analysis

Hematoxylin and eosin (H&E) and immunohistochemical (IHC) staining were performed on 3.5 µm thick serial sections from formalin-fixed paraffin-embedded (FFPE) IH tissues. The primary antibodies employed for the study are shown in Table 2. All reactions were revealed using an UltraView Universal DAB Detection Kit (Ventana, Export, PA, USA) or IHC Detection Kit-Micropolymer (Abcam, Cambridge, UK; ab236466) according to the manufacturer’s recommendations. Light hematoxylin was used to counterstain nuclei. The immunohistochemical analysis of nestin, CD26, and CD133 was repeated at least three times, yielding similar results. CD26 IHC required freshly cut FFPE tissue for optimal detection. Negative controls consisted of sections exposed to the same procedure but omitting primary antibodies.

The evaluation of immunostaining was performed independently by three pathologists (EMS, FPP, and LG) using a light microscope (Olympus BX60, Tokyo, Japan) connected to a digital camera (QICAM; QImaging, Surrey, BC, Canada).

### 4.3. Hemangioma-Derived Endothelial Cell Lines

Proliferative infantile hemangioma-derived endothelial cells (Hem-ECs) were isolated and cultured under sterile conditions as previously described in detail [58]. Briefly, Hem-ECs were cultured in a complete medium consisting of Endothelial Cell Growth Medium (EGM, Mobile, AL, USA; ready-to-use; Cell Applications, Inc., San Diego, CA, USA; 211-500), 100 U/mL penicillin, and 100 μg/mL streptomycin (Gibco; 15140-122), and incubated at 37 °C in a humidified atmosphere containing 5% CO_2_. Cells were tested for Mycoplasma contamination at the beginning and during all experiments using a detection kit according to the manufacturer’s protocol (PCR Mycoplasma Detection Kit; abm, New York, NY, USA; G238).

### 4.4. Immunofluorescence/Immunocytochemistry

As previously described elsewhere [58,59], immunofluorescence assays were performed on isolated Hem-ECs using the following primary unconjugated antibodies: mouse anti-human CD31 (1:50; 30 min; 37 °C; DAKO, Carpinteria, CA, USA, M0823) and rabbit anti-human von Willebrand factor (vWF) (1:200; overnight; 4 °C; DAKO, A0082). Anti-mouse and anti-rabbit IgG FITC-conjugated secondary antibodies (1:70; 60 min; 37 °C; Merck, Rahway, NJ, USA, F4018, and F9887) were employed, respectively. Nuclei were recognized by 4′,6-Diamidino-2-phenylindole (DAPI; 0.5 mg/mL, 15 min at RT; Merck, D8417) counterstaining. Cover slips were mounted with Vectashield (Vector Laboratories, Newark, CA, USA, H-1000) and samples were analyzed by a fluorescence microscope (Olympus BX60) connected to a digital camera (QICAM). GLUT1 was investigated by immunoperoxidase. As above, after fixation and 3% hydrogen peroxide solution for 10 min, chamber slides containing Hem-ECs were incubated with rabbit anti-human GLUT1 (ready to use, 37 °C; 30 min; Ventana-Roche, Basel, Switzerland). The immunoreaction was revealed using an IHC Detection Kit-Micropolymer (Abcam; ab236466) according to the manufacturer’s recommendations. Hematoxylin was used as nuclear counterstain. Following the application of coverslips, slides were analyzed under an optical microscope (Olympus BX60) connected to a QICAM camera.

### 4.5. MTT Assay

Ninety-six-well tissue culture flat-bottom plates (Corning, Corning, NY, USA; 3595) were coated with collagen type 1 solution (Merck; C8919). Hem-ECs were seeded at 4 × 10^3^ cells per well in 100 µL complete medium. After adhesion, Hem-ECs were exposed to various vildagliptin (Selleckchem, Houston, TX, USA; LAF-237) µM concentrations (1, 10, 25, 50, 100, 250, 500, and 1000). Vildagliptin (VLDG) is a DPP-IV inhibitor, clinically approved to treat type 2 diabetes mellitus. After 24 h and 72 h of VLDG exposure, the treatment medium was replaced with 100 µL of 3-(4,5-dimethylthiazol-2-yl)- 2,5-diphenyltetrazolium bromide (MTT) at 1 mg/mL (Merck; M2128). Plates were further incubated at 37 °C for 2 h. At the end of the incubation, the formazan was dissolved using 100 µL of dimethyl sulfoxide (Merck; D2650) per well. The optical density was measured with a microplate reader (VictorTMX4; Perkin Elmer, Waltham, MA, USA). All experiments were carried out three times, and five replicates for each condition were made.

### 4.6. Western Blot Analysis

Sixty µg of proteins from Hem-ECs lysates, untreated or treated with 100 µM and 250 µM VLDG for 24 h, were resolved by SDS-PAGE and transferred to nitrocellulose membranes (Invitrogen, Thermo Fisher Scientific, Waltham, MA, USA; 88018). Membranes were incubated with 1:1000 rabbit anti-human p21^CIP1/WAF1^ (hereafter named p21) (Cell Signaling Technology, Danvers, MA, USA; #2947S) and 1:500 mouse anti-HSP90 (Santa Cruz Biotechnology, Dallas, TX, USA; sc-69703). Membranes were then washed and incubated with anti-mouse or anti-rabbit antibodies at 1:5000 dilution (Invitrogen, Waltham, MA, USA, A32735 Alexa FluorTM Plus 800 IRDye^®^800CW Goat anti-Rabbit IgG (H + L); LI-COR, Biotechnology, #925-68020 IRDye^®^680LT Goat anti-Mouse IgG). Blots were detected using the ChemiDocTM MP Imaging System (Bio-Rad Laboratories, Hercules, CA, USA).

### 4.7. Flow Cytometric Analysis

One hundred µL PBS containing 1 × 10^6^ Hem-ECs were incubated for 20 min in a dark room at 4 °C with 5 µL mouse anti-human CD26-PE (DB Biosciences, Franklin Lakes, NJ, USA; L272). For ki67 staining, at least 1 × 10^6^ Hem-ECs, untreated or treated with 100 µM or 250 µM VLDG for 24 h, were harvested to be subjected to BD Cytofix/Cytoperm™ Fixation/Permeabilization Kit (BD Biosciences; 554714), according to the manufacturer’s protocol. Then, 2 µL anti-human ki67-FITC antibody REAfinity™ (Miltenyi Biotec, Washington, DC, USA; 130-117-691) was added for 30 min in a dark room at 4 °C. The negative controls consisted of cells exposed to the same procedure but omitting the primary antibody.

In all experiments, at least 3 × 10^4^ cells were acquired with an Attune™ NxT Acoustic Focusing Cytometer (Invitrogen, Thermo Fisher Scientific) after forward (FSC-A) and side (SSC-A) scatter gating strategies. All acquisitions were analyzed with FlowJo v10.8 software (BD Biosciences).

### 4.8. Apoptosis Assay and Cell Cycle Analysis

For the apoptosis assay, 1 × 10^5^ Hem-ECs were seeded in 6-well cell culture plates (Greiner, Monroe, NC, USA; 657160), coated with collagen type 1 solution. The next day, the medium was replaced with complete medium in the presence or absence of vildagliptin (100 and 250 µM) for 72 h. Hem-ECs were harvested and stained with eBioscience™ Annexin V Apoptosis Detection Kits (Invitrogen, Thermo Fisher Scientific, BMS500FI-100) according to the manufacturer’s instructions. The apoptosis rate of cells was assessed using an Attune™ NxT Acoustic Focusing Cytometer. A minimum of 20,000 events was collected for each condition.

To perform the cell cycle assay, Hem-ECs were cultured with complete medium in the presence or absence of vildagliptin (100 and 250 µM) for 72 h. Hem-ECs were harvested and fixed with 70% ethanol solution and stained with FxCycle™ PI/RNase Staining Solution (Invitrogen, Thermo Fisher Scientific, F10797) according to the manufacturer’s instructions. DNA contents were measured using an Attune™ NxT Acoustic Focusing Cytometer. A minimum of 15,000 events was collected for each condition. All data were analyzed with FlowJo v10.8 software.

### 4.9. Statistical Analysis

MTT assay data are represented as mean± standard errors (SE) and were analyzed using one-way analysis of variance (ANOVA) with Holm-Sidak’s post hoc test. *p* ≤ 0.05 was always considered significant.

## Figures and Tables

**Table 1:** Summary results of all IH cases.

	N Cases	CD26^pos^ %	CD133^pos^ %
**Proliferative IH**	15	100	66.6
**Involuting IH**	12	58.3	25
*w/o mixed phase*	5	0	0
*w mixed phase*	7	100	42.8

**Table 2:** Primary antibodies used for IHC assay.

Marker	Source	Company	Antigen Retrieval	Processing System Tool	Dilution	Time (min)	Temp. (°C)	Clone
CD31	Ms	Ventana-Roche	15′ MW	*	ready to use	28	37	JC70
GLUT1	Rb	Ventana-Roche	15′ MW	*	ready to use	28	37	polyclonal
D2-40	Ms	Cell Marque	15′ MW	*	ready to use	28	37	D2-40
ki67	Ms	DAKO	15′ MW	*	1:100	28	37	Mib-1
WT1	Ms	Ventana-Roche	15′ MW	*	ready to use	28	37	6F-H2
Nestin	Rb	Millipore	15′ MW	**	1:1000	45	RT	polyclonal
CD26	Rb	Cell Signaling Technology	35′ bath 95 °C	**	1:50	o/n	4	D6D8K
CD133	Rb	Abcam	35′ bath 95 °C	**	1:100	60	RT	polyclonal

* BenchMark Ultra (Ventana-Roche); ** Manual; MW = microwave oven; Ms = mouse; Rb = rabbit; RT = room temperature; o/n = overnight.

## Data Availability

All data of this work are present in the paper.

## Supplementary Materials

The following supporting information can be downloaded at: https://www.mdpi.com/article/10.3390/ijms25189760/s1.
