# Supplementary figures and images for: CD26 Is Differentially Expressed throughout the Life Cycle of Infantile Hemangiomas and Characterizes the Proliferative Phase
Source: Int J Mol Sci. 2024 Sep 10;25(18):9760. doi: 10.3390/ijms25189760 (PMC11432178; doi:10.3390/ijms25189760)

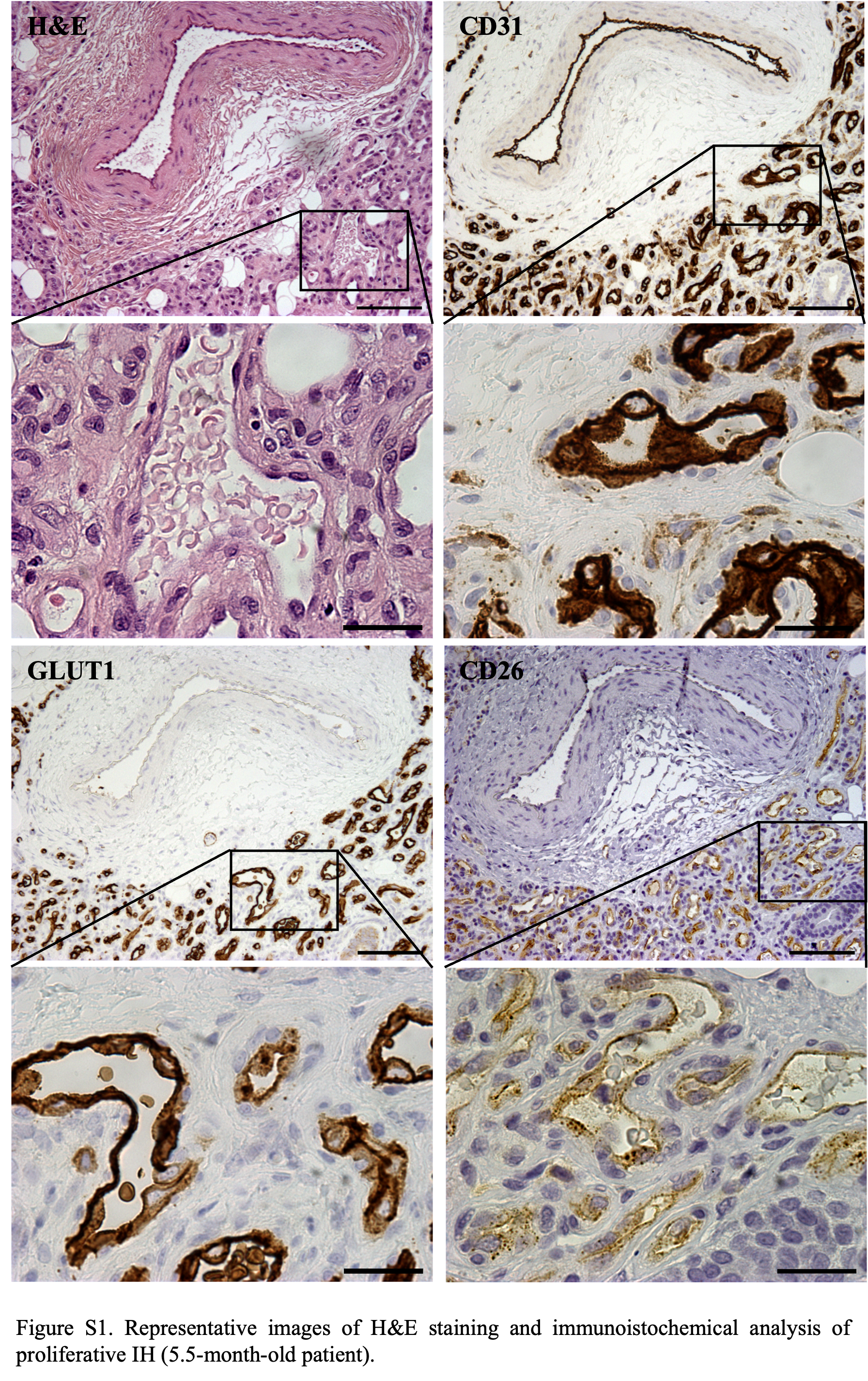

Supplement: Supplementary file 1 [file ijms-25-09760-s001.zip › ijms-3123046-supplementary.png]
